# Increased vulnerability to psychological distress and suicidal ideation among transgender women with self-reported disabilities in San Francisco, California

**DOI:** 10.1016/j.pmedr.2025.103083

**Published:** 2025-04-25

**Authors:** Abtin Shafie, Alexis Salvatore, Riley Shea, Eileen Yu, Oyku Ozyucel, Ansharan Akbar, Bow Suprasert, Erin C. Wilson, Willi McFarland, Kelly D. Taylor, Sean Arayasirikul

**Affiliations:** a University of California Riverside (UCR), 900 University Ave, Riverside, CA 92521, USA; b San Diego State University (SDSU), 5500 Campanile Dr, San Diego, CA 92182, USA; c University of San Francisco (USF), 2130 Fulton Street, San Francisco, CA 94117, USA; d University of California Irvine (UCI), 260 Aldrich Hall, Irvine, CA 92697, USA; e University of California Los Angeles (UCLA), 405 Hilgard Ave, Los Angeles, CA 90095, USA; f University of Maryland (UMD), 7901 Regents Drive, College Park, MD 20742, USA; g Center for Public Health Research, San Francisco Department of Public Health (SF DPH), 25 Van Ness Ave., San Francisco, CA 94102, USA; h Division of Prevention Science, University of California San Francisco (UCSF), 550 16th Street, San Francisco, CA 94158, USA; i The Legacy Center and Department of Health, Society, and Behavior, Joe C. Wen School of Population & Public Health, University of California Irvine (UCI), 856 Health Sciences Quad, Irvine, CA 92697, USA

**Keywords:** Disability, Psychological distress, Transgender women, Health equity, Suicidal ideation

## Abstract

**OBJECTIVE:**

Research among transgender women has found health, but especially mental health, to be shaped by social inequities and multiple, intersectional, structural vulnerabilities. While some studies have shown that transgender adults are also more likely to have a disability than cisgender adults, few studies have explored this intersection. We assess possible associations between disability status and psychological stress and suicidal ideation among transgender women.

**METHODS:**

We analyzed data from the San Francisco site of the National HIV Behavioral Surveillance Transgender (NHBS-Trans) Study (2019–2020) to explore how disability status among transgender women influences mental health. Chi-squared and Fisher's exact tests to assess associations between disability status and psychological distress. Multivariable logistic regression models assessed the magnitude of these associations adjusted for potential confounders.

**RESULTS:**

71.14 % of the sample (*N* = 201) reported living with one or more disabilities. Participants with 1+ disabilities had more than 10 times the odds of reporting high levels of psychological distress (aOR 10.46, 95 % CI 3.06–35.74) and more than five times the odds of reporting suicidal ideation (aOR 5.83, 95 % CI 1.69–20.15) compared to those with no disabilities. Participants with 2+ disabilities had 10+ times the odds of reporting suicidal ideation compared to participants with no disabilities (aOR 10.77, 95 % CI 2.94–39.51).

**CONCLUSIONS:**

Transgender women living with disabilities face multiple intersecting inequities likely attributable to living in a world that is not built for them on the basis of gender identity and disability status. The resulting psychological distress, alongside transphobia and ableism, can exacerbate mental health issues.

## Highlights

• Disability, psychological distress, suicide were assessed in a community sample.
• Disabilities were prevalent and diverse among the sample of transgender women.
• Disability status was associated with psychological distress and suicide ideation.

## 1 Introduction

People with disabilities comprise one of the largest minoritized groups in the United States (U·S). According to 2021 U.S. Census Bureau data, there are about 42.5 million Americans with disabilities (Schaeffer RLaK, 2023). A disability is any condition of the body or mind (impairment) making it more difficult for the person with the condition to do certain activities (activity limitation) and interact with the world around them (participation restrictions) (CDC, 2024a). According to the Center for Disease Control's (CDC) Disability and Health Data System, disability can be assessed based on six functional disability types: cognitive, mobility, hearing, vision, self-care, and independent living (CDC, 2024b). Among U.S. adults, more than one in four have a disability (CDC, 2024a). People with disabilities face greater health inequities, disparities, and poorer health outcomes than nondisabled people (Friedman, 2024; Okoro et al., 2018). The disparities that people with disabilities face are inextricably linked to and exacerbated by social determinants of health (Emerson et al., 2011; Friedman, 2024; Frier et al., 2018). Examples of salient social determinants of health include one's physical and social environments, socioeconomic status, access to and quality of healthcare, and access to and quality of education (Spruce, 2019).

Social determinants of health for people with disabilities are negatively impacted by ableism which can worsen physical and mental health (Emerson et al., 2011). For example, ableism towards people with disabilities exists in society as a lack of accessible housing, transportation, and healthcare providers (Emerson et al., 2011; Frier et al., 2018). The uneven impact of social determinants of health among people with disabilities have also been found to be correlated with gender, age, race, type of disability, and severity of disability (Friedman, 2024). Individuals with disabilities that are part of an additional minoritized group experience greater disparities in health outcomes compared to nondisabled people (Centers for Disease C, Prevention, 2008; Krahn et al., 2015; Wisdom et al., 2010). People with disabilities are more than twice as likely to have comorbid conditions (CDC, 2024a; Kinne et al., 2004; Mills, 2023; WHO, 2024) and less likely to report receiving sufficient social and emotional support, perpetuating comorbidities such as depression and anxiety (Krahn et al., 2015). Moreover, people with disabilities are stigmatized in society, often exacerbating and worsening comorbidities and disabilities (General, 2005).

Disability research with transgender populations is limited. Two studies found that transgender adults are more likely to report living with a disability than cisgender adults (Herman et al., 2017; Smith-Johnson, 2022). Other research has identified that one in five cisgender women worldwide live with a disability, and that cisgender women are more likely to develop a disability than cisgender men due to facing gender-based violence and encountering discrimination in healthcare (Kabir, 2020). Transgender women face similar yet different forms of systemic oppression, stigma, gender-based violence, and discrimination based on racial/ethnic and gender identity (Arayasirikul et al., 2022). Potentially as a result of these intersecting factors, transgender people have disproportionately higher rates of poor mental health compared to cisgender people (Hanna et al., 2019).

Poor mental health outcomes in transgender and gender expansive (TGE) people include psychological distress, depression, and suicidality (Hanna et al., 2019) largely attributed to experiencing victimization in the forms of harassment, physical assault, sexual violence, and chronic stress from persistent stigma and discrimination related to their minoritized identities (Valentine and Shipherd, 2018; White Hughto et al., 2015). Those living with a disability are more likely to report poorer mental health than nondisabled people (General, 2005; Krahn et al., 2015). Few studies have examined the intersection of mental health and disability status in TGE populations. One study found that lesbian, gay, bisexual and transgender (LGBT)-identifying adults with disabilities had higher odds of reporting mental health issues compared to nondisabled non-LGBT-identifying adults, but the study made no distinction between specific gender identities and sexual orientations of participants (Brucker et al., 2023). This study seeks to explore the relationship between disability and mental health use among a population of adult transgender women or people assigned male sex at birth and identify as a gender other than man, women of trans experience, and transfeminine people (hereafter “transgender women” as the term preferred by our study's Community Advisory Board) in San Francisco. While these risks may impact all transgender people, our study focuses on transgender women because of the rigor and availability of relevant data with transgender women. We therefore conducted a secondary analysis of data from the San Francisco site of the first transgender women cycle of the National HIV Behavioral Surveillance (NHBS-Trans) in 2019–2020.

## 2 Methods

### 2.1 Ethics approval

All procedures performed in studies involving human participants were in accordance with the ethical standards of the institutional and/or national research committee and with the 1964 Helsinki declaration and its later amendments or comparable ethical standards. This study protocol was approved by the Institutional Review Board at the University of California, San Francisco (IRB #15–17,775). All participants provided informed consent.

### 2.2 Study design and sample

We conducted a secondary analysis of San Francisco site of the CDC-led NHBS-Trans conducted in 2019–2020. Respondent-driven sampling (RDS), a sampling methodology utilizing peer referrals to engage hardly reached populations, was used to obtain a diverse, community-based sample of transgender women. A diverse set of twenty-five transgender women were identified as the “seeds” who recruited eligible participants in the community from their social networks. Eligibility criteria were: identifying as a transgender woman (i.e., identified as a woman, a transgender woman, or another gender other than male assigned at birth), 18 years or older, and a San Francisco resident. Informed consent was obtained from all individual participants included in the study. For the present study, data originate from an interviewer-administered questionnaire. After completing the study, participants were asked to refer up to ten other transgender women to the study. Participants received $100 for completing study activities and an additional $25 for each eligible peer referral. Recruitment continued until the final sample size was reached and the demographic characteristics of the sample stabilized.

### 2.3 Measures

*Socio-demographics*. Socio-demographic characteristics examined included age, race/ethnicity, education, income, employment status, housing status, and history of incarceration.

*Disability status*. The NHBS-Trans questionnaire measured types of disability by asking participants the following six questions (yes/no): Are you deaf or do you have serious difficulty hearing (hearing)? Are you blind or do you have serious difficulty seeing, even when wearing glasses (vision)? Because of a physical, mental, or emotional condition, do you have serious difficulty concentrating or making decisions (cognitive)? Do you have serious difficulty walking or climbing stairs (ambulatory)? Do you have difficulty dressing or bathing (self-care)? Because of a physical, mental, or emotional condition, do you have difficulty doing errands alone, such as visiting a doctor's office or shopping (independent living)? We constructed three levels of disability status to assess layered disability by summing counts across these six indicators of disability to compare those who did not report a living with a disability to those who are living with one type of disability and to those who are living with two or more types of disabilities.

*Mental Health*. To measure mental health, we include measures for psychological distress and risk of suicide. Psychological distress status over the past 30 days was assessed using the Kessler-6 psychological distress scale (Kessler et al., 2010). The Kessler-6 asks participants to assess the following six items in the past 30 days - (Schaeffer RLaK, 2023) felt nervous, (CDC, 2024a) felt hopeless, (CDC, 2024b) felt restless, (Friedman, 2024) felt depressed, (Okoro et al., 2018) everything was an effort, and (Emerson et al., 2011) felt worthless - none of the time, a little of the time, some of the time, most of the time, and all of the time. We constructed a dichotomous variable with two levels to measure high levels of psychological distress (Kessler-6 score 13–24) and low level of psychological distress (Kessler-6 score 0–12). We measured suicidal ideation, planned suicide and attempted suicide by asking participants the following dichotomous (yes/no) questions: During the past 12 months did you seriously think about trying to kill yourself? During the past 12 months, did you make any plans to kill yourself? During the past 12 months, did you try to kill yourself?

### 2.4 Statistical analysis

We use univariate statistics to describe the study sample and key outcomes, bivariate statistics using chi-squared and Fisher's exact tests to assess the relationship between disability status and mental health outcomes. For any outcomes that were statistically significant at the bivariate level, we built multivariable logistic regression models using a stepwise approach. Multivariable logistic regression models assessed the magnitude of these relationships adjusted for age. All analyses were conducted with STATA statistical software (version 18) (*StataCorp, inventorStata Statistical Software: Relase* 18, 2023). There were no missing data.

## 3 Results

Overall, 201 transgender women participated in the study. Table 1 displays sociodemographic characteristics of the sample. Over one-third (39.3 %) were age 50 years or older. Over one-third were also Hispanic (37.3 %). Regarding level of education, 22.0 % did not have a high school diploma or equivalent degree, while 12.5 % had a Bachelor's degree or higher education. Nearly two-thirds (65.5 %) reported an annual income below $15,000 (approximately the federal poverty level). A plurality of respondents reported that they were unable to work due to health reasons (29.9 %) followed by being unemployed for 12 months or longer (29.4 %). Being currently unhoused was reported by 26.9 %. Two-thirds (67.2 %) of transgender women reported being incarcerated at some point in their lives. A majority of participants (71.9 %) reported any disability. Nearly one-third of transgender women reported one disability (32.3 %) and more than one-third (38.8 %) reported two or more disabilities. The most commonly reported type of disability was cognitive difficulty (52.2 %,) followed by ambulatory difficulty (29.9 %), independent living difficulty (27.9 %), vision difficulty (17.9 %), hearing difficulty (13.9 %), and self-care difficulty (7.0 %).

**Table 1:** Sociodemographic characteristics and self-reported disability status of transgender women, National HIV Behavioral Surveillance, Transgender Women's cycle, San Francisco site, 2019–2020 (*N* = 201).

				Overall
			n	%
***Sociodemographics***				
	Age in years			
		18–29	28	13.9
		30–39	42	20.9
		40–49	52	25.9
		50+	79	39.3
	Race/ethnicity			
		Hispanic	75	37.3
		non-Hispanic Black/African American	42	20.9
		non-Hispanic White	36	17.9
		non-Hispanic Multi	25	12.4
		non-Hispanic Asian	15	7.5
		non-Hispanic American Indian/Alaskan Native, non-Hispanic Native Hawaiian/Pacific Islander	–	3.5
	Education			
		No high school diploma or equivalent degree	44	22.0
		High school diploma or equivalent degree only	62	31.0
		Some college, Associate's degree, or technical degree	69	34.5
		Bachelor's degree or more	25	12.5
	Annual Income			
		Below federal poverty level ($15,000 or less)	131	65.5
		Above federal poverty level (more than $15,000)	69	34.5
	Employment Status			
		Unable to work for health reasons	60	29.9
		Unemployed long-term - 12 months or longer	59	29.4
		Employed full-time (32–40 h/week)	28	13.9
		Unemployed short term - less than 12 months	24	11.9
		Employed part-time (less than 32 h a week)	22	11.0
		Self-employed	14	7.0
		Retired	11	5.5
		Homemaker or Other	–	5.0
	Currently unhoused			
		No	146	72.6
		Yes	54	26.9
	Incarcerated (ever)			
		No	66	32.8
		Yes	135	67.2
***Self-Reported Disability Status***				
	Hearing			
		No	173	86.1
		Yes	28	13.9
	Vision			
		No	165	82.1
		Yes	36	17.9
	Cognitive			
		No	96	47.8
		Yes	105	52.2
	Ambulatory			
		No	141	70.1
		Yes	60	29.9
	Self-care			
		No	187	93.0
		Yes	14	7.0
	Independent living			
		No	145	72.1
		Yes	56	27.9
***Number of disabilities***				
	None		58	28.9
	One		65	32.3
	Two or more		78	38.8

Table 2 reports data on mental health indicators. Fifty-one (25.4 %) participants had Kessler-6 scores that indicate a high level of psychological distress. Overall, 17.4 % of transgender women reported suicidal ideation, 7.5 % reported having planned a suicide, and 5.0 % reported a suicide attempt in the past 12 months.

**Table 2:** Bivariate relationships between self-reported disability status and mental health among transgender women, National HIV Behavioral Surveillance, Transgender Women's cycle, San Francisco site, 2019–2020 (N = 201).

	Overall (*N* = 201)		No disability (*n* = 58)		One or more disability (*n* = 143)		
	n	%	n	%	n	%	p-value^a^
Psychological distress (Kessler-6 score)							
Low (0−12)	150	74.6	55	94.8	95	66.4	0.00
High (Arayasirikul et al., 2022; Brucker et al., 2023; General, 2005; Hanna et al., 2019; Herman et al., 2017; Kabir, 2020; Kessler et al., 2010; Kinne et al., 2004; Mills, 2023; Smith-Johnson, 2022; Valentine and Shipherd, 2018; White Hughto et al., 2015)	51	25.4	–	5.2	48	33.6	
Suicidal Ideation (past 12 months)							
No	166	82.6	55	94.8	111	77.6	0.00
Yes	35	17.4	–	5.2	32	22.4	
Planned Suicide (past 12 months)							
No	186	92.5	57	98.3	129	90.2	0.07
Yes	15	7.5	–	1.7	14	9.8	
Attempted Suicide (past 12 months)							
No	191	95.0	57	98.3	134	93.7	0.29
Yes	10	5.0	–	1.7	9	6.3	

a p-value obtained from chi-share test.

Table 2 also compares mental health outcomes among transgender women with no disabilities to those with one or more disabilities. A greater proportion of participants with one or more disabilities reported suicidal ideation in the past 12 months compared to participants with no disabilities (22.4 % vs 5.2 %, *p* < 0.05). A greater proportion of participants with one or more disabilities reported high levels of psychological distress compared to participants with no disabilities (33.6 % vs 5.2 %, p < 0.05).

**Fig. 1:**
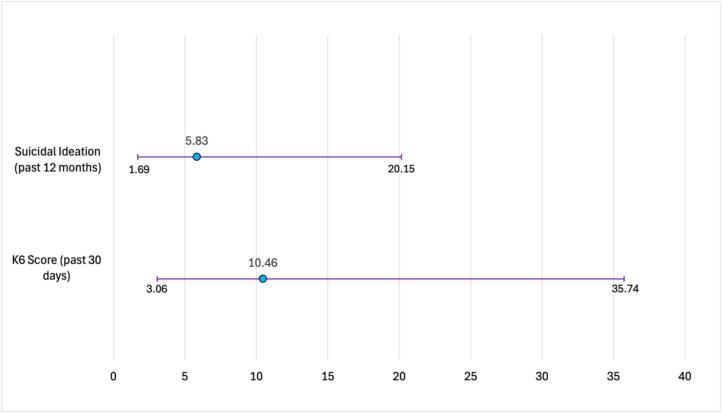
^a^ Psychological distress was measured by the Kessler-6 instrument with a dichotomous variable using a score threshold of 12.

Fig. 1 presents associations with one or more disabilities to those with no disability (referent group) adjusting for age. Participants with one or more disabilities had more than 10 times the odds of reporting high levels of psychological distress compared to those with no disabilities (aOR 10.46, 95 % CI 3.06–35.74). Participants with one or more disabilities had more than five times the odds of reporting suicidal ideation in the past 12 months compared to those with no disabilities (aOR 5.83, 95 % CI 1.69–20.15).

**Fig. 2:**
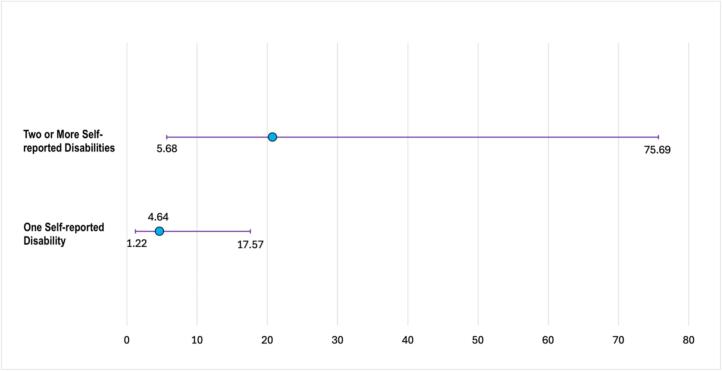
Age-adjusted associations of high psychological distress for transgender women with one, and two or more self-reported disabilities compared to those with no self-reported disability, National HIV Behavioral Surveillance, Transgender Women's cycle, San Francisco site, 2019–2020 (*N* = 201).

**Fig. 3:**
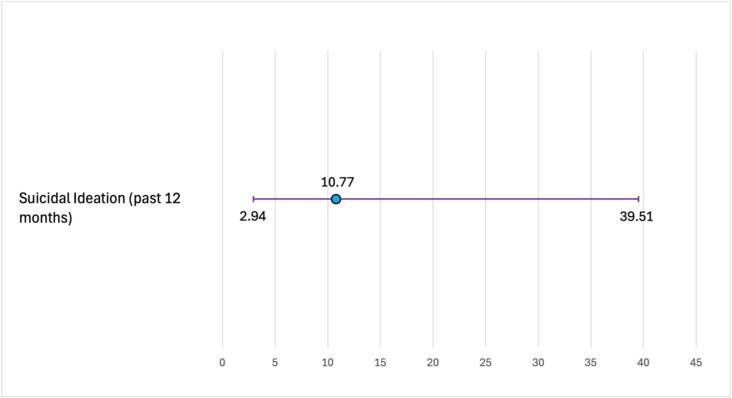
Age-adjusted association of suicidal ideation for transgender women with two or more self-reported disabilities compared to those with no self-reported disability, National HIV Behavioral Surveillance, Transgender Women's cycle, San Francisco site, 2019–2020 (*N* = 201).

Fig. 2 shows associations between high psychological distress for participants with one disability (aOR 4.64, 95 % CI 1.22–17.57) and two or more disabilities (aOR 20.74, 95 % CI 5.68–75.69) compared to those with no disabilities, adjusting for age. Participants with two or more disabilities had over ten times the odds of reporting suicidal ideation compared to participants with no disabilities (aOR 10.77, 95 % CI 2.94–39.51), adjusting for age (Fig. 3).

## 4 Discussion

Our findings show that among transgender women having one or more disabilities is associated with high levels of psychological distress and increased likelihood of reporting suicidal ideation. Our findings contribute to the limited research characterizing disability and mental health among transgender individuals (Smith-Johnson, 2022). The link we found between disability status and mental health may be contextualized by other work that found that people with disabilities more commonly face mental distress, like depression and anxiety, due to lack of social and emotional support (Krahn et al., 2015). This work argues that one's social and environmental contexts perpetuate disparities and inequity that people living with disabilities face – a society that is not designed to meet the needs of people living with disabilities is what needs to change.

We also observed the differential impact of layered disability on poor psychological distress. While people with two or more disabilities and people with one disability compared to those with no disability were more likely to experience psychological distress, we find that there is an amplification effect where people with two or more disabilities are more than five times more likely than people with one disability to report high levels of psychological distress. Recent research has found similar findings among the general population. Marlow et al. found that in a nationally representative sample, suicidal ideation and suicide attempt were significantly more likely among people with multiple disability types (Marlow et al., 2022). This reinforces the need to conceptually frame disability status as dynamic, complex, and multidimensional rather than static and unidimensional.

Similarly, an intersectional approach is critical in furthering research on disability and health among transgender communities. Disability is but one social position among many others, that includes – being transgender, being a woman, being racial/ethnic minoritized person. These factors condition one's opportunities for health and shape how oppression is lived. While health disparities research among populations of transgender women has applied race, as a proxy for racism, as a driver of health inequity, unfortunately disability and ableism are often overlooked (Wickenden, 2023).

Our study is subject to limitations. The sampling method used in this study, RDS, may not have produced a representative sample of transgender women. Transgender women of lower socioeconomic status may have been more inclined to participate than transgender women with higher income given the study incentives for participation and recruitment as well as the demands on their time (Raymond et al., 2017). Data in this study were collected from an interviewer-administered survey and sensitive topics stigmatized in society may be subject to social desirability bias. While the study included measures on disability, the primary objective of the San Francisco site of the NHBS-Trans study is to track behaviors related to HIV in San Francisco. This analysis drew from the San Francisco site of the NHBS-Trans study and not all participating sites. San Francisco is a unique community with historical significance for transgender populations and a more developed public health and social service environment; these data cannot be generalized to other NHBS-Trans sites. As a cross-sectional survey, these data cannot directly address to temporality or assess causation. Disability and its severity measured cross-sectionally at one point in time is likely to increase over time for individuals (Ceolin et al., 2024).

Despite limitations, our findings have implications, in guiding future public health research with transgender populations. Not only is the population of transgender people growing (Twenge et al., 2024), healthy aging across the life course is critical especially as the likelihood of developing one or more disabilities also increases with age (Ceolin et al., 2024). Additionally, transgender people experience intersectional forms of oppression, discrimination, and violence that contribute to chronic stress (Arayasirikul and Wilson, 2019). Recognizing the already elevated strain associated with living with a disability in an inaccessible social world (Mills, 2023), the convergence of these multiple intersectional forms of stress for transgender communities with disabilities merits future investigation. Future research investigating specific pathways and antecedents of mental health among diverse transgender populations and disability are needed. In particular, disability studies that involve transgender men, transgender women, and nonbinary folks will better examine both shared and unique impacts of disability status on health and wellbeing.

Clinicians serving transgender communities living with disabilities ought to consider incorporating assessments for social determinants of health, especially factors in social and environmental domains that may exacerbate inaccessibility and negatively impact their physical and mental health and well-being. For example, housing and employment are areas in which transgender women experience discrimination due to gender oppression and transphobia (Begun and Kattari, 2016). Limited options for housing, working conditions, and employment will undoubtedly reduce the kinds of accommodations transgender women with disabilities may need and deserve as well as lead to poor access to health care (Raiford et al., 2016). These experiences can create chronic daily stress and negative mental and physical health outcomes and even worsen existing disabilities (Bariola et al., 2015). Incorporating standards of care for transgender people with disabilities across the life course is needed (Coleman et al., 2022).

Strengthening and delivering medical education that is knowledgeable about both transgender health and disabilities is needed. Not only do health care providers report limited opportunities to learn about the health of gender and sexual minorities (Bradford et al., 2013), TGE individuals also report a lack knowledgeable physicians and having to educate their physician on their unique health needs (Grant et al., 2011). Similarly, healthcare professionals receive insufficient training in working with patients with disabilities (Iezzoni et al., 2021) and places where care is delivered continue to be inaccessible in a range of ways to patients with various disabilities (Agaronnik et al., 2019). As little as a one-hour lecture on the key concepts of TGE people and the health disparities they face led medical students to feel more comfortable and confident caring for TGE patients (Najor et al., 2020).

## 5 Conclusion

These findings shed light on the relationship between disability status and mental health in transgender women. Our findings suggest that disability status may add an additional layer of chronic stress, compounding the stressors associated with having to navigate a world laden with transphobia and ableism, worsening psychological distress. As the global population ages and the population of transgender people grows, the prevalence of disabilities will increase, and it will be important to advance strategies, interventions, and clinical practices that address chronic stress at this critical intersection.

Age-adjusted associations of mental health outcomes for transgender women with one or more self-reported disability compared to those with no self-reported disability, National HIV Behavioral Surveillance, Transgender Women's cycle, San Francisco site, 2019–2020 (*N* = 201).

## CRediT authorship contribution statement

**Abtin Shafie:** Writing – original draft, Formal analysis, Conceptualization. **Alexis Salvatore:** Writing – review & editing. **Riley Shea:** Writing – review & editing. **Eileen Yu:** Writing – review & editing. **Oyku Ozyucel:** Writing – review & editing. **Ansharan Akbar:** Writing – review & editing. **Bow Suprasert:** Writing – review & editing, Validation, Data curation. **Erin C. Wilson:** Writing – review & editing, Investigation. **Willi McFarland:** Writing – review & editing, Resources, Investigation, Funding acquisition. **Kelly D. Taylor:** Writing – review & editing. **Sean Arayasirikul:** Writing – original draft, Supervision, Resources, Project administration, Formal analysis, Data curation, Conceptualization.

## Funding

This work was supported by the 10.13039/100000030Centers for Disease Control and Prevention (CDC)’s 10.13039/100005258National Center for HIV/AIDS, Viral Hepatitis, STD, and TB Prevention under grant [number 6NU62ES005077] and the 10.13039/100000025National Institute of Mental Health (NIMH) under grant [number R25MH119858]. This study's funding sources had no role in the study design; in the collection, analysis, and interpretation of data; in the writing of the report; or in the decision to submit the article for publication.

## Declaration of competing interest

The authors declare the following financial interests/personal relationships which may be considered as potential competing interests:All authors report financial support was provided by National Institute of Mental Health. Willi McFarland reports financial support was provided by Centers for Disease Control and Prevention. If there are other authors, they declare that they have no known competing financial interests or personal relationships that could have appeared to influence the work reported in this paper.

## Data Availability

The data that has been used is confidential.
